# Predicting ionizing radiation exposure using biochemically-inspired genomic machine learning

**DOI:** 10.12688/f1000research.14048.2

**Published:** 2018-06-15

**Authors:** Jonathan Z.L. Zhao, Eliseos J. Mucaki, Peter K. Rogan

**Affiliations:** 1 Department of Biochemistry, Schulich School of Medicine and Dentistry, Western University, London, ON, N6A 2C1, Canada; 2 Department of Computer Science, Faculty of Science, Western University, London, ON, N6A 2C1, Canada; 3 Department of Epidemiology & Biostatistics, Schulich School of Medicine and Dentistry, Western University, London, ON, N6A 2C1, Canada; 4 CytoGnomix Inc., London, ON, N5X 3X5, Canada; 5 Department of Oncology, Schulich School of Medicine and Dentistry, Western University, London, ON, N6A 2C1, Canada

**Keywords:** Ionizing Radiation Exposure, Machine Learning, Gene Signatures, Molecular Diagnostics, Validation, Biodosimetry, Support Vector Machine, Minimum Redundancy Maximum Relevance

## Abstract

**Background:** Gene signatures derived from transcriptomic data using machine learning methods have shown promise for biodosimetry testing. These signatures may not be sufficiently robust for large scale testing, as their performance has not been adequately validated on external, independent datasets. The present study develops human and murine signatures with biochemically-inspired machine learning that are strictly validated using k-fold and traditional approaches.

**Methods:** Gene Expression Omnibus (GEO) datasets of exposed human and murine lymphocytes were preprocessed via nearest neighbor imputation and expression of genes implicated in the literature to be responsive to radiation exposure (n=998) were then ranked by Minimum Redundancy Maximum Relevance (mRMR). Optimal signatures were derived by backward, complete, and forward sequential feature selection using Support Vector Machines (SVM), and validated using k-fold or traditional validation on independent datasets.

**Results:** The best human signatures we derived exhibit k-fold validation accuracies of up to 98% ( *DDB2*, *PRKDC*, *TPP2*, *PTPRE*, and *GADD45A*) when validated over 209 samples and traditional validation accuracies of up to 92% ( *DDB2*, *CD8A*, *TALDO1*, *PCNA*, *EIF4G2*, *LCN2*, *CDKN1A*, *PRKCH*, *ENO1*,  and *PPM1D*) when validated over 85 samples. Some human signatures are specific enough to differentiate between chemotherapy and radiotherapy. Certain multi-class murine signatures have sufficient granularity in dose estimation to inform eligibility for cytokine therapy (assuming these signatures could be translated to humans). We compiled a list of the most frequently appearing genes in the top 20 human and mouse signatures. More frequently appearing genes among an ensemble of signatures may indicate greater impact of these genes on the performance of individual signatures. Several genes in the signatures we derived are present in previously proposed signatures.

**Conclusions:** Gene signatures for ionizing radiation exposure derived by machine learning have low error rates in externally validated, independent datasets, and exhibit high specificity and granularity for dose estimation.

## Introduction

Potential radiation exposures from industrial nuclear accidents, military incidents, or terrorism are threats to public health ^1^. There is a need for large scale biodosimetry testing, which requires efficient screening techniques to differentiate exposed individuals from non-exposed individuals and to determine the severity of exposure ^2^. Current diagnostic techniques, including the cytogenetic gold standard ^3– 6^, may require several days to provide accurate dose estimates ^1, 7^ of large cohorts. To address the need for faster diagnostic techniques that accurately measure radiation exposures, gene signatures based on transcriptomic data have been introduced ^7– 10^. Probit regression models of radiation response using 25 probes on peripheral blood samples achieved up to 90% accuracy for distinguishing between irradiated blood samples and unirradiated controls ^9^. A 74-gene classifier based on nearest centroid expression levels was 98% accurate in distinguishing four levels of irradiation from controls ^10^. This level of performance implies that samples exposed to different levels of radiation may be distinguishable based on mRNA expression levels of different genes. While this suggests the feasibility of transcriptional modeling of radiation responses, validation with external datasets is required to establish its reliability for rapid diagnostics. A caveat of these signatures is that they have not all been externally validated on datasets independent of the source data used for model development. A 29-gene signature modelled using a support vector machine (SVM) was externally validated on such a dataset, resulting in 80% accuracy in distinguishing higher (≥8Gy) from lower dose (≤2Gy) radiation exposure in novel samples ^7^. Previous studies have identified biomarkers that distinguish irradiated ( *ex vivo*) from unirradiated blood samples with high accuracies ^11– 15^. The present study derives signatures with improved performance on externally validated samples by employing a different selection of modelling techniques. The machine learning pipeline used here addresses some of the previous limitations through a more rigorous feature selection process and stricter validation procedures.

Previously, the Student’s t-test ^7^, the F-test ^10^, and correlation coefficients ^9^ were used to identify potential radiation biomarker genes. Although statistical criteria can distinguish genes that are differentially expressed upon radiation exposure, they do not eliminate expressed genes with redundant responses to radiation exposure. Redundancy increases the possibility of overfitting, thereby reducing the generalizability of these models to predict responses in independent datasets. We address this limitation with the information theory-based criterion for gene selection known as minimum redundancy maximum relevance (mRMR) ^16– 18^, which ranks genes according to shared mutual information between expression levels and radiation dose (relevance), and by minimizing mutual information shared by expression values of these and other genes (redundancy) ^17, 18^. mRMR outperforms ranking criteria based solely on maximizing relevance ^17^. In contrast with heuristic approaches like differential expression, we only consider genes with evidence of a relationship to radiation response, which significantly limits the number of model features. Biochemically-inspired genomic machine learning (ML) has been used to derive high performing gene signatures that predict chemotherapy and hormone therapy responses ^18– 20^. From an initial set of mRMR-derived biochemically relevant genes, wrapper approaches for feature selection ^21^ are used to find an optimal set of genes that predict exposure to radiation.

It can be challenging to obtain highly accurate models that perform well on externally validated samples for several reasons. Aside from biases in training data, batch effects and lack of reproducibility may introduce systematic and random sources of variability into gene expression microarray data. Different source datasets can impact data normalization, reducing model performance. We utilize two validation procedures. The first is a signature-centric approach that mirrors external k-fold validation ^7^. The limitation of signature-centric validation is that, while signatures allow for the identification of important genes associated with radiation response, a tangible model is required to generate actual diagnostic predictions. To address this limitation, we also use a second model-centric approach, which we term “traditional validation”. This procedure applies quantile normalization to training and test data before a model is fitted to the training data. This quantile method has been shown to be more effective than scaling, loess, contrast, and non-linear methods in reducing variation between microarray data ^22^. Model validation was not expected to perform as well as signature validation, because quantile normalization is not always successful in eliminating variation between microarray datasets, whereas k-fold validation is independent of this source of variation. This study shows that robust model validation is a critical step in reproducibly predicting which individuals have been exposed to significant levels of radiation.

## Methods

### Datasets

Murine gene expression datasets ^23^ were obtained from peripheral blood (PB) mononuclear cell samples of ten-week old C57B16 mice that either received total body radiation at 50 cGy, 200 cGy, or 1000 cGy or were not exposed. Post-exposure, total RNA was isolated after 6 hours and expression was determined by microarray analysis using Operon Mouse V3.0.1 (Gene Expression Omnibus (GEO): GPL4783 from GSE10640[GPL4783]) ^24^ and Operon Mouse V4.0 arrays (GEO: GPL6524 from GSE10640[GPL6524]) ^24^. Similar analyses were performed with human expression microarrays ^18^, including datasets GEO: GSE6874[GPL4782] ^9^, GSE10640[GPL6522] ^24^, GSE1725 ^25^, and GSE701 ^26^. GSE6874 and GSE10640 consist of PB samples collected 6 hours post-exposure from healthy donors and patients undergoing total body irradiation at 150–200 cGy analyzed with Operon Human V3.0.2 (GEO: GPL4782) and Operon Human V4.0 (GEO: GPL6522) microarrays. GSE10640[GPL6522] consists of 32 patients treated with alkylator-based chemotherapy without radiation. GSE1725 contains lymphoblastoid cell line samples derived from 57 subjects treated with 500 cGy. RNA was extracted 4 hours after exposure. Expression was measured using Affymetrix Human Genome U95 Version 2 Array (GEO: GPL8300). GSE701 contains lymphoblastoid cell lines from Fondation Jean Dausset-CEPH which were irradiated at 300 cGy or 1000 cGy and extracted 1–24 hours after exposure. Expression was measured using the Affymetrix Human Genome U95A Array (GEO: GPL91). The GSE77254 dataset ^27^ was also used to validate our human signatures. This dataset consisted of blood samples collected from baboons that were either total body or partial body irradiated with Cobalt 60 at either 2.5 or 5 Gy. Expression for each subject was measured 1 to 2 days after exposure and was related to their hematologic acute radiation syndrome (HARS) scores.

### Preprocessing ( Figure 1, panel i)

Rows and columns of microarray data that are less than 95% complete were removed and any remaining missing values were imputed using the nearest-neighbor algorithm. Only genes that are common across all datasets have been retained. Expression values of each probe were transformed to z-scores and the mean expression value of probes for the same gene have been assigned as the expression of each gene. Human and murine signatures were derived separately.

**Figure 1.**
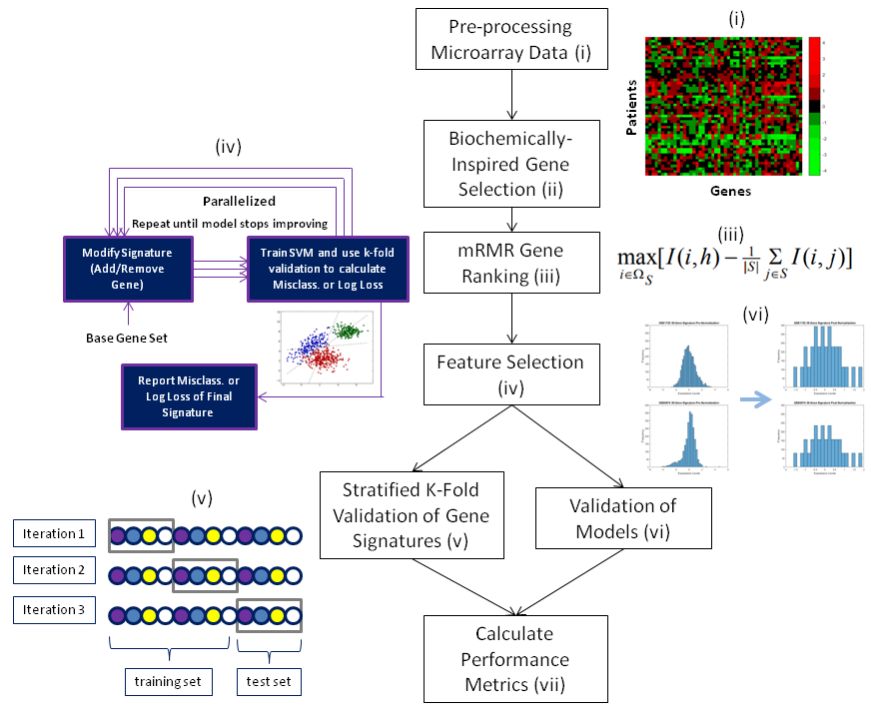
Flow chart of the biochemically inspired machine learning pipeline used to derive gene signatures. In panel (v), k-fold validation splits data into k sections, where each section acts as a test set in turn while the remaining sections act as a training set. Panel (v) depicts k-fold validation for *k* = 3. Coloured circles represent the samples in a dataset where different colours represent different radiation doses. In panel (vi), quantile normalization forces data into the same distribution. To demonstrate this, thirty random genes were chosen to form a signature. The histograms on the left represent the distributions of expression levels of these genes in the pre-normalized datasets GSE1725 and GSE6874[GPL4782]. The histograms on the right represent the distribution of expression levels of the same genes post-normalization.

### Biochemically-inspired gene selection ^18– 20^ ( Figure 1, panel ii)

A literature search has been conducted to identify genes implicated in radiation response using the search queries “radiation genes,” “radiation response genes,” and “radiation signatures” on PubMed. Cited genes comprise those differentially expressed after radiation exposure, genes present in DNA repair databases and other radiation signatures, and evolutionarily conserved genes that were highly expressed in radio-resistant species. A list of 998 genes was compiled ^28– 41^, Supplementary Table X) for deriving signatures.

### Minimum Redundancy Maximum Relevance (mRMR) gene ranking ^11, 12^ ( Figure 1, panel iii)

Rank is assigned by incremental selection of genes based on the mutual information difference (MID) criterion ^16, 17^. Highly ranked genes have expression information that shares mutual information with radiation exposure and shares little information with expression of other genes. The MID criterion used to select the next ranked gene is ${\text{max}}_{\text{i}\in \Omega }\left[I(i,h)-\frac{1}{\left|S\right|}\sum_{j\in S}I(i,j)\right],$ where *i* is a gene selected from *Ω*, the total gene space, *S* is the set of genes selected before *i*, | *S*| is the number of genes selected before *i*, *I*( *i*, *h*) is the mutual information between expression of gene *i* and radiation dose ( *h*), and *I*( *i*, *j*) is the mutual information between expression of gene *i* and expression of gene *j*.

### Support Vector Machine (SVM) Learning

SVM models are classifiers that use hyperplane boundaries to separate samples into exposure classes by maximizing the distance between the separating hyperplanes and samples of each class. The *fitcecoc* function of MATLAB 2017a’s Statistics and Machine Learning Toolbox ^42^ with a SVM template was used to fit SVM models to training data. The *fitcecoc* function was used because it allows the fitting of multiclass models, which was required for analysis of murine samples that were irradiated at four different exposure levels. The SVM models use the Gaussian radial basis function kernel and a range of selected box-constraint and kernel-scale parameters. The box-constraint, denoted by the variable *C*, determines how severely misclassifications are penalized during training. The kernel-scale, denoted *σ*, represents the width of the Gaussian radial basis function. These parameters collectively control the tradeoff between underfitting and overfitting ^43^. After feature selection, a grid search is performed to determine the optimal ( *C*, *σ*) combination for values of *C* and *σ* between 1 and 100000 (inclusive) by powers of 10 such that *C* ≥ *σ*.

### Feature selection (FS) ( Figure 1, panel iv) ^21^

**Figure 2.**
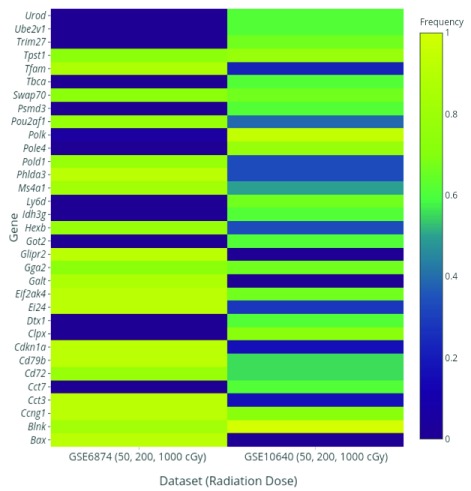
Heat map depicting the gene compositions of the top 20 murine signatures derived from different datasets: GSE6874[GPL4783] and GSE10640[GPL6524]. Gene frequency values are first scaled within datasets and then scaled across datasets to ensure values between 0 and 1.

**Figure 3.**
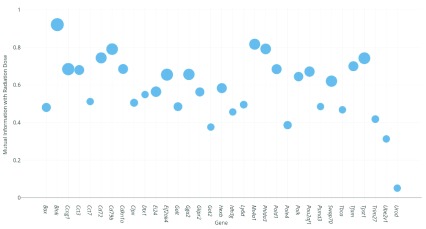
Scatter plot depicting the mutual information each gene’s expression shares with radiation exposure (averaged over GSE6874[GPL4783] and GSE10640[GPL6524]). The size of each circle is proportional to the frequency at which the gene appears in the top 20 murine signatures ranked by log loss averaged over GSE6874[GPL4783] and GSE10640[GPL6524]. The genes presented match those of the Figure 2.

Greedy feature selection was used to derive signatures. Complete sequential feature selection (CSFS) sequentially adds genes to an initially empty base set. The added gene is the highest mRMR-ranked gene that is not already included. This is repeated until all genes have been evaluated and the best performing subset of genes is identified. Forward sequential feature selection (FSFS) sequentially adds genes from the top 50 mRMR ranked genes to an initially empty base set. The added gene is the one whose addition improves the model by the greatest margin. Backward sequential feature selection (BSFS) sequentially removes genes from the top 30 mRMR ranked genes. The gene removed is the one whose removal causes the greatest improvement in the model. For BSFS and FSFS, we measure model improvement using misclassification or log loss during k-fold validation (see *Performance metrics* section below). Genes are added or removed until model performance plateaus. During feature selection, *C* and *σ* parameters need to be chosen for SVM learning (see *SVM Learning* section above). Thus, each signature is characterized by the feature selection algorithm used, the dataset used to derive it, and the *C*- *σ* combination used for its SVM models during feature selection. This leads to a large number of possible signatures (see Supplementary Files Y1–Y7). Supplementary Files Y1–Y3 and Supplementary Files Y6–Y7 contain k-fold validation results from which the top 20 signatures (evaluated using average validation log loss), in particular, were analyzed ( Figure 2, Figure 3, Figure 6, Figure 7).

### Validating signatures ( Figure 1, panel v)

Stratified k-fold validation was used to validate signatures. Samples of the validation dataset were partitioned into *k* sets, comprised of an approximately equal distribution of radiation levels. For validation, each set was used to test a model trained on the remaining sets, resulting in predictions for all samples in the dataset. Advantages of this approach are that variation between datasets is not pertinent and that signatures can be validated on differently labeled datasets (with samples irradiated at different levels).

### Validating models ( Figure 1, panel vi)

Model validation requires separate training and test datasets (the training set is often used for FS). Genes from the signature are extracted from the training and test sets and their expression values are quantile normalized by sample. An important distinction between our approach and a previous study ^7^ is that quantile normalization is applied immediately before validation, so expression of only the genes present in the signature being validated have been normalized. By contrast, previous approaches perform quantile normalization over entire datasets; while this reduces variability in expression values within datasets, it also suppresses the dynamic range, with potential consequential effects on the prognostic value of expression data. After normalization, an SVM model was fit to training datasets and used to generate predictions from the test dataset.

### Performance metrics ( Figure 1, panel vii)

Performance was determined by comparing predicted radiation doses with actual radiation exposures of each sample. Metrics included misclassification error rate, goodness-of-fit, and multi-class log loss. Misclassification is the percentage of samples that were incorrectly classified, goodness-of-fit is the average absolute value difference between predicted radiation exposure and actual radiation exposure, and multi-class log loss is $-\frac{1}{N}\sum_{i=1}^{N}\sum_{j=1}^{M}y_{ij}\text{ln}p_{ij}$ where *N* is the number of samples, *M* is the number of class labels, *p*_*ij*_ is the predicted probability that observation *i* is in class *j*, and *y*_*ij*_ is an indicator variable equal to 1 if sample *i* is in class *j* and 0 otherwise.

## Results

We discovered radiation gene signatures using the microarray data of human and mouse peripheral blood samples and human lymphoblastoid cell lines, which were validated either according to signature ( Figure 1, panel v) or with the respective model ( Figure 1, panel vi). The murine data were obtained from a wider range of radiation exposure levels (0 cGy, 50 cGy, 200 cGy, 1000 cGy) than the human whole body radiation datasets, which were binary comparisons of radiation effects (0 cGy vs. 150-200 cGy, 0 cGy vs. 500 cGy, or 300 cGy vs. 700 cGy). This made possible the discovery of murine gene signatures with finer granularity for discriminating individuals exposed to different exposure levels, which is not currently feasible with the human samples.

### Murine gene signatures

Table 1 displays the murine signatures derived using our pipeline which had the best performance metrics during k-fold validation on an independent dataset. In addition to the signature information, we report the feature selection algorithm ( FS Algorithm) used to discover the signature, the internal validation performance metrics ( FS Misclassification fraction and FS Log Loss function). Validation performance metrics on external dataset(s) are indicated by the Validation Misclassification fraction, Validation Log Loss function, and Validation goodness of fit or ( GoF). In the FS Misclass. and FS Log Loss columns, one value is always N/A because signatures are derived by optimizing either misclassification or log loss, but never both. The remaining murine signatures are presented in Supplementary Files Y6 and Supplementary Files Y7.

**Table 1.** Best murine signatures assessed by K-Fold validation.

Signature ( *C, σ*)	FS ^1^ Algo.	FS ^1^ Misclass.	FS ^1^ Log Loss	Validation Misclass.	Validation Log Loss	Validation GoF ^2^
**a) Derived from GSE6874[GPL4783] and 5-fold Validated on GSE10640[GPL6524] (n = 75)**						
*Phlda3 Blnk Bax Cdkn1a Cct3 Pold1 Cd79b Ei24* *Eif2ak4 Ccng1 Glipr2 Hexb Pou2af1 Swap70* *Apex1 Ptpn1 Mdm2 Tpst1 Ly6e Sdcbp* (10, 10)	BSFS	N/A	0.08	0.08 ± 0.00	0.29 ± 0.02	15 ± 0
*Phlda3 Blnk Bax Cdkn1a Cct3 Tfam Pold1 Cd72* *Cd79b Ei24 Galt Eif2ak4 Ms4a1 Ccng1 Glipr2* *Gga2 Sh3bp5 Hexb Gcdh Pou2af1 Swap70 Apex1* *Ptpn1 Mdm2 Tpst1 Ly6e Sdcbp Lcn2 Suclg2* (100000, 100)	BSFS	0.04	N/A	0.10 ± 0.00	0.23 ± 0.01	26 ± 1
*Cdkn1a Blnk Phlda3 Sdcbp Ccng1* (1000, 100)	FSFS	N/A	0.13	0.17 ± 0.00	0.49 ± 0.01	12 ± 0
**b) Derived from GSE10640[GPL6524] and 6-fold Validated on GSE6874[GPL4783] (n = 103)**						
*Blnk Ccng1 Tpst1 Pole4 Eif2ak4 Atp5l* (100000, 100)	FSFS	N/A	0.12	0.11 ± 0.00	0.35 ± 0.01	25 ± 0
*Blnk Polk Sod3 Ube2v1 Eif2ak4* (10000, 100)	FSFS	N/A	0.22	0.20 ± 0.00	0.64 ± 0.01	18 ± 0

^1^FS: Feature Selection ^2^GoF: Goodness of Fit.

A list of the most consistently appearing genes in the best performing signatures were obtained by pooling the top 20 murine signatures (assessed by validation log loss) from GSE6874[GPL4783] and GSE10640[GPL6524], and respectively collating the top 17 and 19 most frequent genes. The union of these two sets comprises 33 genes displayed in a heat map based on the frequencies of each gene ( Figure 2). Surprisingly, the compositions of signatures derived from both datasets are not as similar as one may expect. The genes that appear more frequently in signatures derived from one dataset infrequently appear in the other even though both datasets consisted of the same types of samples irradiated at the same exposure levels.

The shared mutual information of these expressed genes with radiation dose ( Figure 3) indicates whether only high mutual information genes appear in the best signatures or whether some lower mutual information genes may also be selected by our feature selection algorithms. The frequency of each gene among these signatures (represented by diameter of the circle) correlates with the mutual information between expression and radiation dose ( *ρ* = 0.8016). However, it would be an oversimplification to create signatures based solely upon mutual information, since some genes in lower performing signatures exhibit higher mutual information content. Development of accurate signatures requires more than a collection of gene features whose individual expression values share information with radiation dose, since many of these genes may reveal similar information, and redundant machine learning model features. For instance, *Bax* and *Blnk* are both common among the best murine signatures, even though *Blnk* shares much more mutual information with radiation dose than *Bax* expression. Since *Blnk* and *Bax* are involved in completely different pathways – *Bax* is an inducer of apoptosis ^44^ whereas *Blnk* is involved in a B-cell antigen receptor signaling pathway required for optimal B-cell development ^45^, they provide different types of information to the overall model. Conversely, we also observe that genes with high information content, such as *Ms4a1*, may appear less frequently than genes with lower information content, such as *Eif2ak4* or *Ccng1*.

Although mRMR prioritizes genes with non-redundant, complementary contributions, subsequent wrapper steps of forward and backward sequential feature selection occur independently of the mRMR ranking. mRMR reduces the list of features considered by these algorithms, but it is possible for only high mutual information genes to be selected for the final signature. Thus, the inclusion of lower mutual information genes, such as *Ube2v1* and *Urod*, reinforces the effectiveness of the mRMR method.

**Figure 4.**
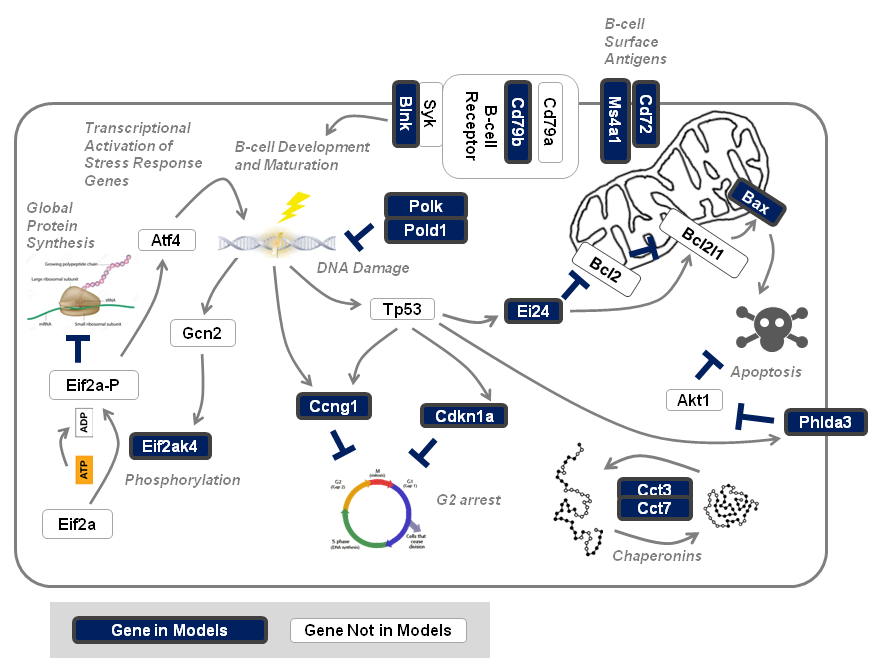
Depiction of the major cellular functions of most frequently appearing genes of the best murine signatures (same genes presented in Figure 1 and Figure 2).

The cellular roles of these protein products ( Figure 2 and Figure 3) demonstrate a variety of pathways and functions ( Figure 4), some of which have previously discussed ^46^. These include DNA repair genes ( *Polk* ^29^ and *Pold1* ^32^), inducers of apoptosis ( *Ei24* ^36^, *Bax* ^36^, and *Phlda3* ^36^), chaperonins ( *Cct3* ^28^ and *Cct7* ^28^), cell cycle regulators ( *Ccng1* ^33^ and *Cdkn1a* ^36^), B-cell development genes ( *Cd79b* ^24^ and *Blnk* ^24^), B-cell antigens ( *Cd72* ^9^ and *Ms4a1* ^24^), and a stress-response kinase that inhibits protein synthesis globally ( *Eif2ak4* ^31^).

**Figure 5.**
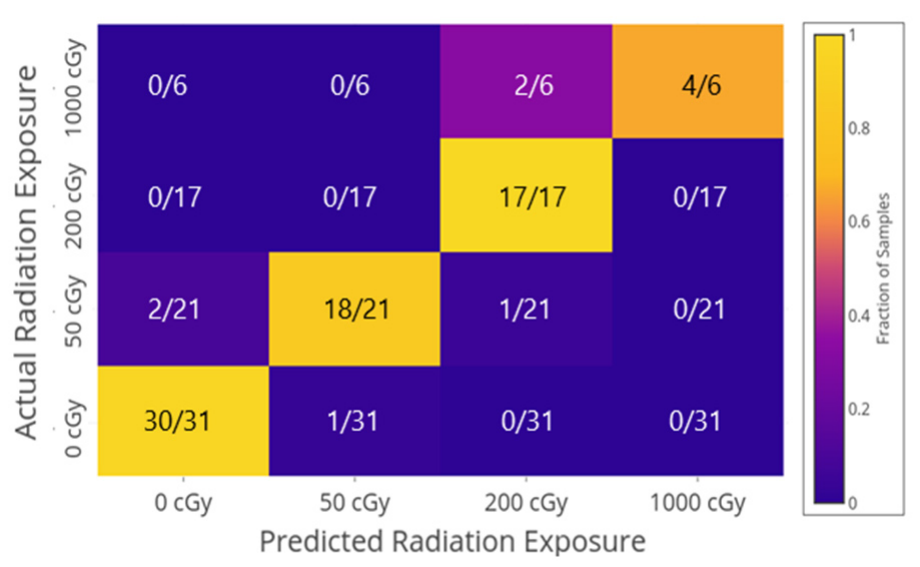
Confusion matrix for murine signature: *Phlda3*, *Bax*, *Cdkn1a*, *Cct3*, *Tfam*, *Pold1*, *Cd72*, *Cd79b*, *Ei24*, *Galt*, *Eif2ak4*, *Ms4a1*, *Ccng1*, *Glipr2*, *Gga2*, *Sh3bp5*, *Hexb*, *Gcdh*, *Pou2af1*, *Swap70*, *Apex1*, *Ptpn1*, *Mdm2*, *Tpst1*, *Ly6e*, *Sdcbp*, *Lcn2*, *Suclg2*. Numerators represent the number of samples in each category while denominators represent the total number of samples that were irradiated at a given exposure level (i.e. is the sum of the number of samples in each row).

One of the best murine signatures derived from GSE10640[GPL4783]: *Phlda3*, *Blnk*, *Bax*, *Cdkn1a*, *Cct3*, *Pold1*, *Cd79b*, *Ei24*, *Eif2ak4*, *Ccng1*, *Glipr2*, *Hexb*, *Pou2af1*, *Swap70*, *Apex1*, *Ptpn1*, *Mdm2*, *Tpst1*, *Ly6e*, *Sdcbp* consistently achieved <10% misclassification error with SVM parameters *C* = 10, *σ* = 10. However, for samples that are incorrectly classified according to this signature, the misclassification percentage does not reveal the actual deviation from the correct dose. The confusion matrix visualizes the prediction accuracy of this signature on GEO: GSE10640[GPL6524] ( Figure 5). Indeed, the performance of the matrix shows that the predicted errors for a small fraction of samples deviate from the actual exposures by no more than a single adjacent exposure level. Although the predictions presented in the confusion matrix come from a single iteration of k-fold validation, the standard error associated with misclassification for this signature is extremely low (0.0013) so this confusion matrix is representative of nearly all possible iterations of k-fold validation.

### Human gene signatures

The best performing signatures obtained from each human dataset, assessed by k-fold validation, are presented in Table 2. Although four human radiation datasets were available, GSE701 contained only 10 samples, which was insufficient for derivation of a unique gene signature. While k-fold validation removes the requirement for inter-dataset normalization, it assesses the ability of signatures (genes) to predict radiation exposure without tying the signatures to corresponding models. Each signature is characterized by the feature selection algorithm and its validation statistics, which have been averaged over the 3 independent datasets that were excluded from the original data used to derive the signature.

**Table 2.** Best human signatures assessed by K-Fold validation.

Signature ( *C*, **)	FS Algo.	Average Misclass.	Average Log Loss	Average GoF
**a) Derived from GSE1725 and K-Fold Validated on GSE10640[GPL6522] (n = 85),** **GSE6874[GPL4782] (n = 78), and GSE701 (n = 10)**				
*GADD45A DDB2* (1, 1)	FSFS	0.07	0.40	24
*PPM1D DDB2 CCNF CDKN1A PCNA GADD45A PRKAB1 TOB1 TNFRSF10B MYC* *CCNB2 PTP4A1 BAX CCNA2 ATF3 LIG1 CCNG1 FHL2 PPP1R2 MBD4 RASGRP2* *UBC NINJ1 TRIM22 IL2RB TP53BP1 PTPRCAP EEF1D PTPRE RAD23B EIF2B4 STX11* *PTPN6 STK10 PSMD1 BTG3 MLH1 RNPEP HSPD1 UNG PTPRC PTPRA BCL2 GSS* *SH3BP5 TPP2 IDH3B CCNH STK11 EIF4EBP2 HSPA4 FADS2 RPA3 GZMK ANXA4* *ICAM1 PPID LMO2 PPIE NUDT1 FUS POLR2A LY9 RPA1 PTS TNFRSF4 RPA2 PSMD8* *GCDH MAN2C1 PTPN2 RUVBL1 ATP5H GK CD79B MAP4K4 POLE3 PRKCH AKT2* *MOAP1 CCNG2 ALDOA SRD5A1 HAT1 XRCC1 EIF2S3 RAD1 UBE2A ZFP36L1 CD8A* *TALDO1 GPX4 SSBP2 ERCC3 ATP5O PEPD EIF4G2 ACO2 HEXB UBE3A ARPC1A* *PSMD10 PRCP PPIB ZNF337 CETN2 RPL29* (10000, 10000)	CSFS	0.07	0.18	14
**b) Derived from GSE10640[GPL6522] and K-Fold Validated on GSE1725 (n = 114),** **GSE6874[GPL4782] (n = 78), and GSE701 (n = 10)**				
*DDB2 RAD17 PSMD9 LY9 PPIH PCNA MDH2 MOAP1 TP53BP1 PPM1D ATP5G1* *BCL2L2 ENO2 PTP4A1 PSMD8 LIG1 FDPS OGDH CCNG1 PSMD1* (100, 100)	BSFS	0.05	0.39	15
*DDB2 HSPD1 ICAM1 PTP4A1 GTF3A LY9* (100000, 10000)	FSFS	0.08	0.16	43
*RAD17 TNFRSF10B PSMD9 LY9 PPIH PCNA ZNF337 MDH2 TP53BP1 PPM1D* *ZFP36L1 ATP5G1 ALDOA BCL2L2 ENO2 GADD45A PTP4A1 PSMD8 LIG1 ATP5O* *FDPS OGDH PSMD1* (10000, 10000)	BSFS	0.05	0.22	11
**c) Derived from GSE6874[GPL4782] and K-Fold Validated on GSE1725 (n = 114),** **GSE10640[GPL6522] (n = 85), and GSE701 (n = 10)**				
*DDB2 PRKDC PRKCH IGJ* (100000, 10000)	FSFS	0.02	0.27	7
*DDB2 PRKDC TPP2 PTPRE GADD45A* (1000, 100)	FSFS	0.02	0.07	5

Since traditional validation typically requires separate training and test sets that feature samples irradiated at the same exposure levels, only signatures derived from GEO: GSE6874[GPL4782] and GEO: GSE10640[GPL6522] could be analyzed. Table 3 presents the best human signatures according to this validation approach. This type of external validation is the most challenging due to the variability associated with different microarray experiments and batch effects of different platforms. This potentially explains the lower performance obtained by traditional validation ( Table 3) compared with k-fold validation on the same datasets ( Table 2). The remaining human signatures are described in Supplementary Files Y1–Y5.

**Table 3.** Best performing human signatures assessed by traditional validation.

Signature ( *C*, σ)	FS Algo.	FS Misclass.	FS Log Loss	Validation Misclass.	Validation Log Loss	Validation GoF
**a) Derived from GSE10640[GPL6522] and Validated on GSE6874[GPL4782] (n = 78)**						
*DDB2 HSPD1 MAP4K4 GTF3A PCNA MDH2* (1000, 10)	FSFS	N/A	2.0E-14	0.14 ± 0.00	0.70 ± 0.03	25 ± 0
*DDB2 GTF3A TNFRSF10B* (1, 1)			0.07	0.20 ± 0.03	0.51 ± 0.00	35 ± 5
**b) Derived from GSE6874[GPL4782] and Validated on GSE10640[GPL6522] (n = 85)**						
*DDB2 CD8A TALDO1 PCNA EIF4G2 LCN2* *CDKN1A PRKCH ENO1 PPM1D* (10000, 1)	BSFS	N/A	0.42	0.08 ± 0.00	0.41 ± 0.00	14 ± 0
*DDB2 CD8A TALDO1 PCNA LCN2 CDKN1A* *PRKCH ENO1 GTF3A IL2RB NINJ1 BAX TRIM22* *PRKDC GADD45A MOAP1 ARPC1B LY9 LMO2* *STX11 TPP2 CCNG1 GABARAP BCL2 GSS* *FTH1* (10000, 1000)		0.08	N/A	0.12 ± 0.00	0.31 ± 0.00	21 ± 0

**Figure 6.**
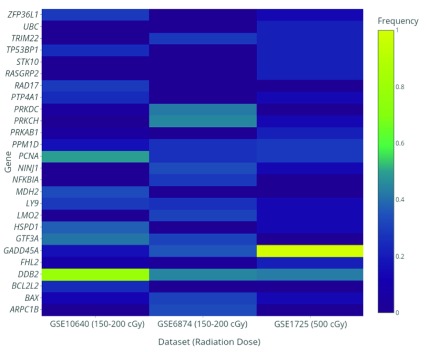
Heat map depicting the gene compositions of the top 20 human signatures derived at different radiation doses: 150-200 cGy (GSE10640[GPL6522], GSE6874[GPL4782]) and 500 cGy (GSE1725). Frequencies are first scaled within and then between datasets to ensure values between 0 and 1.

**Figure 7.**
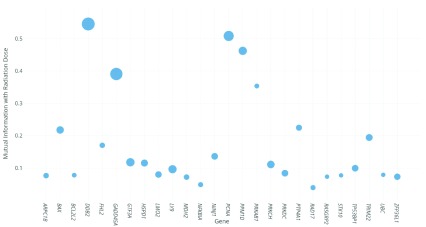
Scatter plot depicting the mutual information each gene’s expression shares with radiation exposure (averaged over GSE10640[GPL6522], GSE6874[GPL4782], and GSE1725). The size of each circle is proportional to the frequency at which the gene appears in the top 20 human signatures ranked by average validation log loss from GSE10640[GPL6522], GSE6874[GPL4782], and GSE1725. The genes shown are also are the same as those indicated in Figure 6.

To determine which human genes are most consistently selected, the most frequently appearing genes (11 or 12 depending on number of equally prevalent genes in different signatures) were compiled from the top 20 human signatures (assessed by lowest average log loss during k-fold validation) from GSE10640[GPL6522], GSE6874[GPL4782], and GSE1725. The union of these three lists indicates the relative frequencies of each gene ( Figure 6). Figure 7 visualizes the mutual information of gene expression ( Figure 6) shared with radiation dose.

While most genes have similar representation in signatures derived from different datasets, *GADD45A* and *DDB2*, in particular, are significantly more frequent in those derived from GSE1725 and GSE10640[GPL6522]. *GADD45A* and *DDB2* are present in signatures derived from samples irradiated at different exposures ( *GADD45A* – 500 cGy, *DDB2* – 150-200 cGy). This raises questions as to whether these genes have a larger influence on the accuracy of individual signatures and whether their expression is calibrated to radiation exposure levels. Removal of these gene features was performed to address their impact. Genes of interest have been removed from each of the top 20 human signatures derived from various datasets and then the signatures were revalidated excluding these features ( Table 4). The difference between the validation metrics preceding and following removal of a gene represents the weight of the gene within a signature. Δ *MC*, Δ *LL*, and Δ *GoF* represent the changes in misclassification, log loss, and goodness of fit, respectively.

**Table 4.** Effect of removing genes from signatures of different datasets.

GSE1725 Validation (0 vs 500 cGy)			GSE10640 Validation (0 vs 150-200 cGy)			GSE6874 Validation (0 vs 150-200 cGy)			GSE701 Validation (300 vs 700 cGy)			Average		
∆ *MC*	∆ *LL*	∆ *GoF*	∆ *MC*	∆ *LL*	∆ *GoF*	∆ *MC*	∆ *LL*	∆ *GoF*	∆ *MC*	∆ *LL*	∆ *GoF*	∆ *MC*	∆ *LL*	∆ *GoF*
**a) Removal of *GADD45A* from signatures derived from GSE1725**														
0.446	0.008	N/A *	0.367	0.373	61.1	0.111	0.561	19.4	0.353	0.529	247	0.319	0.368	109
**b) Removal of *GADD45A* from signatures derived from GSE10640[GPL6522]**														
0.001	0.011	0.658	0.001	0.237	N/A *	-0.007	0.008	-1.29	0.043	1.45	29.8	0.010	0.427	9.72
**c) Removal of *DDB2* from signatures derived from GSE10640[GPL6522]**														
0.128	0.166	64.2	0.078	0.211	N/A *	0.103	0.157	17.9	0	0.471	0	0.08	0.251	27.4
**d) Removal of *DDB2* from signatures derived from GSE1725**														
0.012	0.044	N/A *	0.069	0.367	0.102	0.153	0.202	0.269	0.003	0.715	2	0.059	0.332	0.790
**e) Removal of *BAX* (control for *GADD45A*) from signatures derived from GSE1725**														
N/A **	0.08	N/A *	0.024	0.478	0.989	0.025	0.025	4.37	0.005	0.006	3.5	0.018	0.147	2.95
**f) Removal of *PRKAB1* (control for *DDB2*) from signatures derived from GSE10640[GPL6522]**														
N/A **	0.001	N/A *	0.011	0.048	5.70	-0.01	0.01	-2.47	0.02	-0.04	14	0.007	0.005	5.74

* *∆*GoF is always N/A for the dataset used to derive signatures because GoF is never used as the optimized metric during signature development (see Feature Selection Algorithms section under Methods). **Unavailable because the top 20 human signatures derived from GSE1725 were all obtained by optimizing log loss rather than misclassification.

*GADD45A* appears in 14 of the top 20 signatures derived from GSE1725. Of the 14 signatures, 10 were single gene signatures, as *GADD45A* alone was expected to sufficiently distinguish irradiated from unirradiated samples. In these cases, it was assumed that a null signature would perform as well as a predictor that randomly draws predictions from a uniform distribution of doses. Removal of *GADD45A* from these 14 signatures, results in an average increase in misclassification, log loss, and goodness of fit by 0.319, 0.368, and 109 cGy, respectively (see Table 4a). In contrast, elimination of *BAX*, which only appears in 2 of the top 20 signatures derived from GSE1725 and results in an average increase in misclassification, log loss, and goodness of fit by 0.018, 0.147, and 2.95 cGy respectively ( Table 4e). Comparing the effects of removing *DDB2* ( Table 4c) and *PRKAB1* ( Table 4f) from the top 20 GSE10640[GPL6522] signatures confirms the impact of genes that frequently occur within the most accurate gene signatures.

However, the diagnostic contributions of *GADD45A* and *DDB2* expression to the radiation levels at which samples were exposed (500 cGy and 150-200 cGy respectively) are confounding. The effects on model performance resulting from removal of *GADD45A* from the GSE10640[GPL6522] signatures ( Table 4b) versus the GSE1725 signatures ( Table 4a) are discordant. Δ *MC* is higher when *GADD45A* is removed from GSE1725, but Δ *LL* is higher when *GADD45A* is removed from GSE10640[GPL6522]. Δ *LL* is large when *GADD45A* is removed from both datasets, which is consistent with the importance of *GADD45A* at both radiation doses. Indeed, *GADD45A* expression has been demonstrated to be rapidly induced by radiation levels as low as 2 Gy ^47^. Similar discordance was observed in the feature removal experiments of *DDB2* ( Table 4c, 4d).

**Figure 8.**
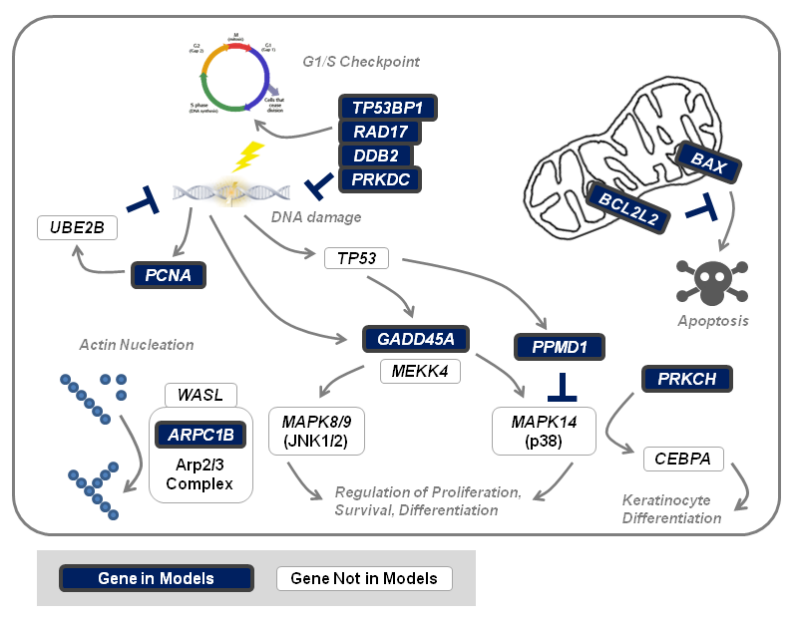
Depiction of major cellular functions of most frequently appearing genes of the best human signatures (see Figure 5 and Figure 6).

As was the case with murine signatures, genes appearing in the best human signatures do not necessarily share high mutual information with radiation dose. However, the compositions of the human signatures are dominated by four genes, *DDB2*, *GADD45A*, *PCNA*, and *PPM1D*, which all share a lot of information with radiation dose ( *DDB2*: 0.55, *GADD45A*: 0.39, *PCNA*: 0.51, *PPM1D*: 0.46). The functions associated with these and less frequently appearing genes are depicted in Figure 8 ^46^. The pathways and functions represented include keratinocyte differentiation ( *PRKCH* ^9^), induction of apoptosis ( *BCL2L* ^37^ and *BAX* ^36^), DNA repair ( *TP53BP1* ^29^, *RAD17* ^30^, *DDB2* ^24^, *PRKDC* ^29^, and *PCNA* ^33^), actin nucleation ( *ARPC1B* ^28^), and regulation of JNK-p38 ( *MAPK14*) signalling ( *GADD45A* ^33^ and *PPMD1* ^33^). The four common genes belong to the DNA repair and regulating JNK-p38 ( *MAPK14*) pathways, which may imply particular significance to these functions in human response to radiation exposure. Interestingly, GADD45A and PPMD1 are antagonistic, that is, GADD45A activates while PPMD1 inhibits p38.

### Validating gene signatures on partial body irradiated samples

We also evaluated the total body irradiation human signatures with expression data from baboons (GSE77254) that were exposed to partial body irradiation. All signatures derived from human samples (see Supplementary Files Y4 and Y5) were completely contained in this dataset and so were eligible for validation. The signatures chosen contained all datapoints, circumventing the need to perform nearest neighbour imputation. Paralogous baboon genes were cross-referenced with those that were used to derive human signatures and expression values of multiple probes within the same gene were averaged.

Signatures were used to differentiate between various label combinations: (1) unirradiated vs. 1 day post-irradiation, (2) unirradiated vs. 2 day post-irradiation, (3) 1 vs. 2 day post-irradiation, (4) unirradiated vs. 1° and 2° HARS, (5) unirradiated vs. 2° and 3° HARS, and (6) 1° and 2° HARS vs. 2° and 3° HARS. Supplementary Files Z1 and Z2 contain validation results based on baboon expression data with human signatures.

Multiple Y4 signatures achieved 0% misclassification in distinguishing unirradiated samples from radiated samples (above label combinations 1, 2, 4, and 5) and multiple Y5 signatures achieved 0% misclassification in label combinations 1, 2, and 5. However, the best performing signatures on this dataset were not the best performing signatures obtained during validation on GSE6874 (Y4) and GSE10640 (Y5). We speculate that technical factors involved in the study design explain why signatures performed differently. For example, the human signatures were derived from blood samples that were collected 6–24 hours after exposure whereas the baboon blood samples were obtained 24–48 hours after exposure. Also, a different microarray platform was used to obtain expression values for the baboon samples.

We also investigated total body radiation signatures on predicting exposures with different sources of partial body irradiation expression data: GSE66372 ^48^ and GSE84898 ^49^. These murine and baboon datasets lacked several genes present in the signatures we derived. None of the Y4 and Y5 signatures were completely contained in GSE66372; the *PSMD9* single gene signature was the only human signature that was completely contained in GSE84898. However, the *PSMD9* signature has poor performance among Y5 signatures based on its log loss metric on GSE6874.

## Discussion

Biochemically inspired genomic signatures of human and murine radiation response exhibit high accuracies in validating independent datasets (98% in k-fold validation, 92% by traditional methods). Some of the human signatures exhibit among the highest specificities reported (e.g. the signature *DDB2*, *CD8A*, *TALDO1*, *PCNA*, *EIF4G2*, *LCN2*, *CDKN1A*, *PRKCH*, *ENO1*, *PPM1D*) exhibited 92% accuracy when validated on GSE10640[GPL6522]. This dataset contains both radiation therapy patients (150–200 cGy) and controls (0 cGy) which include healthy donors and chemotherapy patients treated with alkylators ^9^. Thus, the signature distinguished radiation-induced and chemotherapy-associated DNA damage.

Some of the best performing signatures consisted of one to three gene features. The first signature in Table 2 contains *GADD45A* and *DDB2*, and exhibits a misclassification error rate of 7%. These relatively short signatures have certain advantages over longer signatures with similar performance. It is more likely that the model can be generalized to a wider spectrum of data, when fewer features are required, and from a practical standpoint, diagnostic tests based on fewer gene expression measurements are less susceptible to experimental error.

*BAX*, an inducer of apoptosis, was the single gene shared among those frequently appearing in both murine and human signatures. One possible explanation for this is that the mouse datasets featured samples irradiated at four levels while human datasets contained samples irradiated at two levels. Genes selected by multi-class model algorithms may better discriminate radiation dose. Nonetheless, the radiation response pathways of mice are not necessarily similar to those of humans. In fact, Lucas *et al*. have shown that the murine signatures they developed are not translatable to human samples ^50^. Furthermore, only two genes, including *BAX*, are shared by the human and murine signatures derived by Dressman *et al*. ^50^.

None of the samples exposed to ≥200 cGy are misclassified below this radiation dose based on the multi-class murine signatures ( Figure 5). In the future, a similar analyses could be performed in clinical studies of human subjects exposed to different radiation levels, which might prove useful for determining treatment eligibility after exposure to high levels of myelosuppressive radiation ^51^.

A comparison of the most frequently appearing genes in the optimal human ( Figure 6) and mouse signatures ( Figure 2) with signatures previously derived in other studies reveals little overlap ( Table 5). The compositional differences can be attributed to types of samples used for model training, microarray platforms used, and feature selection techniques used in deriving signatures. However, genes consistently selected in optimized signatures in at least three independent studies include *BAX*, *DDB2*, *GADD45A*, *LY9*, and *TRIM22*. Expression of these genes is indeed predictive of radiation dose and not a result of noise in individual datasets. An ensemble signature consisting of these genes achieves up to 92.3% accuracy in k-fold validation over 277 samples and up to 81.2% accuracy in traditional validation over 78 samples. The quality of the gene signature is largely determined by the quality and amount of training data used to fit the SVM model. Thus, this level of accuracy is not the upper bound on the performance of an SVM of the ensemble signature. Additional data at exposures with fixed levels of radiation in matched training and testing samples could improve model performance.

**Table 5.** Genes found in best performing signatures and previously derived signatures.

Prior Studies	Validation Performance			Shared Genes in Signatures
	K-Fold (internal)	K-Fold (external)	Traditional (external)	
Dressman *et al*. (human) ^9^	90%	N/A	N/A	*BAX*, *DDB2*, *PRKCH*
Dressman *et al*. (mouse) ^9^	N/A	N/A	N/A	*Bax*, *Cd72*, *Cd79b*, *Cdkn1a*, *Ei24*, *Galt*, *Glipr2*, *Ly6d*, *Ms4a1*, *Tfam*
Paul *et al*. (human) ^10^	98%	N/A	N/A	*BAX*, *DDB2*, *GADD45A*, *LY9*, *PCNA*, *PPM1D*, *PTP4A1*, *RASGRP2*, *TRIM22*
Lu *et al*. (human) ^7^	~90%	86%	N/A	*DDB2*, *FHL2*, *GADD45A*, *LY9*, *TRIM22*
This study (human)	100%	98%	92%	N/A
This study (mouse)	99%	92%	N/A	N/A

Ensemble models should be considered which combine genes discovered in different well-performing signatures. Although the most frequently represented human and murine genes were compiled, genes common to one dataset did not appear equally frequently in signatures from the other. This discordance may possibly result of noise in the different datasets, or perhaps to intrinsic differences between them. Compilation of frequently appearing genes in different datasets may be useful for discovery of consistently represented genes that are incorporated into high-performance signatures.

The types of data available for this study and the analytical approaches we used potentially limited the interpretation of these gene signatures. Blood samples of mouse and human datasets were all collected within 24 hours of exposure. Thus, signatures derived on these datasets may only be valid in white blood cells with a limited time window (<24 hours). Additionally, one of the datasets we used to derive signatures, GSE6874, appears to have been a particularly noisy dataset, based on the average misclassification rates on GSE10640, GSE1725, and GSE6874 of 0.03, 0.02, and 0.11, respectively. Assuming that it is possible to differentiate samples irradiated at different levels of exposure using expression data, the feature selection misclassification metric estimates the theoretical limit of how well differentially irradiated samples can be separated based on expression. The surprisingly high feature selection misclassification values obtained from GSE6874 may therefore be indicative of greater levels of noise in the data. Lastly, the greedy feature selection algorithms used to derive signatures cannot guarantee optimal results, that is, we cannot confirm that we have found the best possible signatures from each dataset for predicting radiation exposure. This potentially explains the discordance in gene composition between murine datasets ( Figure 2).

Nevertheless, the validation performance of radiation signatures is significantly improved ( Table 5). The signatures that were externally k-fold validated achieved nearly 100% accuracy. Some of our human signature models are also externally validated in the traditional sense (i.e. using a single model). This validation method, which is representative of an actual scenario, achieves >90% accuracy, and is directly relevant to creating a routine, efficient and highly accurate expression-based radiation prognostic assay.

## Data availability

The data referenced by this article are under copyright with the following copyright statement: Copyright: © 2018 Zhao JZL et al.

Data associated with the article are available under the terms of the Creative Commons Zero "No rights reserved" data waiver (CC0 1.0 Public domain dedication). 

All data underlying the results are available as part of the article and no additional source data are required.

ZENODO: Matlab code for “Predicting Exposure to Ionizing Radiation by Biochemically-Inspired Genomic Machine Learning”, doi: 10.5281/zenodo.1170572 ^52^

Code is available under the terms of the Creative Commons Attribution 4.0 International license (CC-BY 4.0).

## Supplementary material

**Supplementary File X: This spreadsheet lists all the genes found from our literature search (see Methods) that were considered during feature selection.** For each gene, we report the reason for inclusion and a link to the paper containing the supporting evidence.

**Supplementary Files Y1–Y7: These files contain information concerning all the total body radiation signatures derived for this paper.** Each file contains the validation results of signatures derived from a particular dataset. Files Y1-Y3 contain the k-fold validation results of human signatures derived from GSE1725, GSE6874, and GSE10640, respectively, while Y4-Y5 contain the traditional validation results of human signatures derived from GSE6874 and GSE10640, respectively. Files Y6-Y7 contain the k-fold validation results of mouse signatures derived from GSE10640[GPL4783] and GSE10640[GPL6524], respectively. Each supplementary file contains the following columns: Signature, FS Algorithm, C, sigma, FS Misclassification, FS Log Loss, K, Misclassification, Misclassification Error, Log Loss, Log Loss Error, Goodness of Fit, and Goodness of Fit Error. These headings are described in the tab titled “Legend” in Files Y1-Y7. In addition, Files Y1-Y3 have three extra columns: Average Misclassification, Average Log Loss, and Average Goodness of Fit, which represent the misclassification, log loss, and goodness of fit, respectively, averaged over all validation sets.

**Supplementary Files Z1–Z2: These files contain results concerning tradiation validation of Y4 and Y5 human signatures on partial body radiation exposed primates.** Different comparison groups described in the text are indicated in separate tabs in each File. Table headings correspond to performance metrics shown for signatures Y4 and Y5.

## References

[1] Pandey BN Kumar A Tiwari P: Radiobiological basis in management of accidental radiation exposure. *Int J Radiat Biol.* 2010;86(8):613–35. 10.3109/09553001003746059 20673129

[2] Sproull MT Camphausen KA Koblentz GD: Biodosimetry: A Future Tool for Medical Management of Radiological Emergencies. *Health Secur.* 2017;15(6):599–610. 10.1089/hs.2017.0050 29193982 PMC5734138

[3] Liu J Li Y Wilkins R: Accurate cytogenetic biodosimetry through automated dicentric chromosome curation and metaphase cell selection [version 1; referees: 2 approved]. *F1000Res.* 2017;6:1396. 10.12688/f1000research.12226.1 29026522 PMC5583746

[4] Rogan PK Li Y Wilkins RC: Radiation Dose Estimation by Automated Cytogenetic Biodosimetry. *Radiat Prot Dosimetry.* 2016;172(1–3):207–17. 10.1093/rpd/ncw161 27412514

[5] Rogan PK Li Y Wickramasinghe A: Automating dicentric chromosome detection from cytogenetic biodosimetry data. *Radiat Prot Dosimetry.* 2014;159(1–4):95–104. 10.1093/rpd/ncu133 24757176 PMC4067226

[6] Shirley B Li Y Knoll JHM: Expedited Radiation Biodosimetry by Automated Dicentric Chromosome Identification (ADCI) and Dose Estimation. *J Vis Exp.* 2017; (127):e56245. 10.3791/56245 28892030 PMC5619684

[7] Lu TP Hsu YY Lai LC: Identification of gene expression biomarkers for predicting radiation exposure. *Sci Rep.* 2014;4:6293. 10.1038/srep06293 25189756 PMC4155333

[8] Ding LH Park S Peyton M: Distinct transcriptome profiles identified in normal human bronchial epithelial cells after exposure to γ-rays and different elemental particles of high Z and energy. *BMC Genomics.* 2013;14:372. 10.1186/1471-2164-14-372 23724988 PMC3680091

[9] Dressman HK Muramoto GG Chao NJ: Gene expression signatures that predict radiation exposure in mice and humans. *PLoS Med.* 2007;4(4):e106, [cited 2018 Jan 12]. 10.1371/journal.pmed.0040106 17407386 PMC1845155

[10] Paul S Amundson SA: Development of gene expression signatures for practical radiation biodosimetry. *Int J Radiat Oncol Biol Phys.* 2008;71(4):1236–44. 10.1016/j.ijrobp.2008.03.043 18572087 PMC2478639

[11] Boldt S Knops K Kriehuber R: A frequency-based gene selection method to identify robust biomarkers for radiation dose prediction. *Int J Radiat Biol.* 2012;88(3):267–76. 10.3109/09553002.2012.638358 22233095

[12] Budworth H Snijders AM Marchetti F: DNA repair and cell cycle biomarkers of radiation exposure and inflammation stress in human blood. *PLoS One.* 2012;7(11):e48619. 10.1371/journal.pone.0048619 23144912 PMC3492462

[13] Knops K Boldt S Wolkenhauer O: Gene expression in low- and high-dose-irradiated human peripheral blood lymphocytes: possible applications for biodosimetry. *Radiat Res.* 2012;178(4):304–12. 10.1667/RR2913.1 22954392

[14] Ghandhi SA Smilenov LB Elliston CD: Radiation dose-rate effects on gene expression for human biodosimetry. *BMC Med Genomics.* 2015;8:22. 10.1186/s12920-015-0097-x 25963628 PMC4472181

[15] Hall J Jeggo PA West C: Ionizing radiation biomarkers in epidemiological studies - An update. *Mutat Res.* 2017;771:59–84. 10.1016/j.mrrev.2017.01.001 28342453

[16] Ding C Peng H: Minimum redundancy feature selection from microarray gene expression data. *J Bioinform Comput Biol.* 2005;3(2):185–205. 10.1109/CSB.2003.1227396 15852500

[17] Peng H Long F Ding C: Feature selection based on mutual information: criteria of max-dependency, max-relevance, and min-redundancy. *IEEE Trans Pattern Anal Mach Intell.* 2005;27(8):1226–38. 10.1109/TPAMI.2005.159 16119262

[18] Mucaki EJ Baranova K Pham HQ: Predicting Outcomes of Hormone and Chemotherapy in the Molecular Taxonomy of Breast Cancer International Consortium (METABRIC) Study by Biochemically-inspired Machine Learning [version 3; referees: 2 approved]. *F1000Res. * 2016;5:2124. 10.12688/f1000research.9417.3 28620450 PMC5461908

[19] Dorman SN Baranova K Knoll JH: Genomic signatures for paclitaxel and gemcitabine resistance in breast cancer derived by machine learning. *Mol Oncol.* 2016;10(1):85–100. 10.1016/j.molonc.2015.07.006 26372358 PMC5528934

[20] Mucaki EJ Zhao JZL Lizotte D: Predicting Response to Platin Chemotherapy Agents with Biochemically-inspired Machine Learning. *bioRxiv.* 2017;231712 10.1101/231712 PMC6329797 30652029

[21] Guyon I Elisseeff A: An Introduction to Variable and Feature Selection. *J Mach Learn Res.* 2003;3:1157–82. Reference Source

[22] Bolstad BM Irizarry RA Astrand M: A comparison of normalization methods for high density oligonucleotide array data based on variance and bias. *Bioinformatics.* 2003;19(2):185–93. 10.1093/bioinformatics/19.2.185 12538238

[23] Edgar R Domrachev M Lash AE: Gene Expression Omnibus: NCBI gene expression and hybridization array data repository. *Nucleic Acids Res.* 2002;30(1):207–10. 10.1093/nar/30.1.207 11752295 PMC99122

[24] Meadows SK Dressman HK Muramoto GG: Gene expression signatures of radiation response are specific, durable and accurate in mice and humans. *PLoS One.* 2008;3(4):e1912, [cited 2018 Jan 12]. 10.1371/journal.pone.0001912 18382685 PMC2271127

[25] Rieger KE Hong WJ Tusher VG: Toxicity from radiation therapy associated with abnormal transcriptional responses to DNA damage. *Proc Natl Acad Sci U S A.* 2004;101(17):6635–40. 10.1073/pnas.0307761101 15096622 PMC404097

[26] Jen KY Cheung VG: Transcriptional response of lymphoblastoid cells to ionizing radiation. *Genome Res.* 2003;13(9):2092–100. 10.1101/gr.1240103 12915489 PMC403696

[27] Port M Hérodin F Valente M: Gene expression signature for early prediction of late occurring pancytopenia in irradiated baboons. *Ann Hematol.* 2017;96(5):859–70. 10.1007/s00277-017-2952-7 28236054 PMC5371629

[28] Grynberg P Passos-Silva DG Mourão Mde M: *Trypanosoma cruzi* gene expression in response to gamma radiation. *PLoS One.* 2012;7(1):e29596. 10.1371/journal.pone.0029596 22247781 PMC3256153

[29] Wood RD Mitchell M Sgouros J: Human DNA repair genes. *Science.* 2001;291(5507):1284–9. 10.1126/science.1056154 11181991

[30] Birrell GW Giaever G Chu AM: A genome-wide screen in *Saccharomyces cerevisiae* for genes affecting UV radiation sensitivity. *Proc Natl Acad Sci U S A.* 2001;98(22):12608–13. 10.1073/pnas.231366398 11606770 PMC60101

[31] Karlin S Mrazek J: Predicted highly expressed and putative alien genes of *Deinococcus radiodurans* and implications for resistance to ionizing radiation damage. *Proc Natl Acad Sci U S A.* 2001;98(9):5240–5. 10.1073/pnas.081077598 11296249 PMC33194

[32] Chistiakov DA Voronova NV Chistiakov PA: Genetic variations in DNA repair genes, radiosensitivity to cancer and susceptibility to acute tissue reactions in radiotherapy-treated cancer patients. *Acta Oncol.* 2008;47(5):809–24. 10.1080/02841860801885969 18568480

[33] Kabacik S Mackay A Tamber N: Gene expression following ionising radiation: identification of biomarkers for dose estimation and prediction of individual response. *Int J Radiat Biol.* 2011;87(2):115–29. 10.3109/09553002.2010.519424 21067298

[34] Zhou LJ Zhu ZH Liu ZX: Identification and transcriptional profiling of differentially expressed genes associated with response to UVA radiation in *Drosophila melanogaster* (Diptera: Drosophilidae). *Environ Entomol.* 2013;42(5):1110–7. 10.1603/EN12319 24331622

[35] Wang LJ Zhou LJ Zhu ZH: Differential temporal expression profiles of *heat shock protein* genes in *Drosophila melanogaster* (Diptera: Drosophilidae) under ultraviolet A radiation stress. *Environ Entomol.* 2014;43(5):1427–34. 10.1603/EN13240 25259697

[36] Chauhan V Howland M Wilkins R: Identification of gene-based responses in human blood cells exposed to alpha particle radiation. *BMC Med Genomics.* 2014;7:43. 10.1186/1755-8794-7-43 25017500 PMC4128605

[37] Dom G Tarabichi M Unger K: A gene expression signature distinguishes normal tissues of sporadic and radiation-induced papillary thyroid carcinomas. *Br J Cancer.* 2012;107(6):994–1000. 10.1038/bjc.2012.302 22828612 PMC3464765

[38] Milanowska K Krwawicz J Papaj G: REPAIRtoire--a database of DNA repair pathways. *Nucleic Acids Res.* 2011;39(Database issue):D788–792. 10.1093/nar/gkq1087 21051355 PMC3013684

[39] Tarrade S Bhardwaj T Flegal M: Histone H2AX Is Involved in FoxO3a-Mediated Transcriptional Responses to Ionizing Radiation to Maintain Genome Stability. *Int J Mol Sci.* 2015;16(12):29996–30014. 10.3390/ijms161226216 26694365 PMC4691159

[40] Mothersill C O’Malley K Harney J: Further investigation of the response of human uroepithelium to low doses of cobalt-60 gamma radiation. *Radiat Res.* 1997;147(2):156–65. 10.2307/3579416 9008207

[41] Lin JY Mühlmann-Diaz MC Stackhouse MA: An ionizing radiation-sensitive CHO mutant cell line: irs-20. IV. Genetic complementation, V(D)J recombination and the scid phenotype. *Radiat Res.* 1997;147(2):166–71. 10.2307/3579417 9008208

[42] MATLAB: Statistics and Machine Learning Toolbox.[cited 2018 Jan 12]. Reference Source

[43] Eitrich T Lang B: Efficient optimization of support vector machine learning parameters for unbalanced datasets. *J Comput Appl Math.* 2006;196(2):425–36. 10.1016/j.cam.2005.09.009

[44] Pawlowski J Kraft AS: Bax-induced apoptotic cell death. *Proc Natl Acad Sci U S A.* 2000;97(2):529–31. 10.1073/pnas.97.2.529 10639111 PMC33959

[45] Jin G Hamaguchi Y Matsushita T: B-cell linker protein expression contributes to controlling allergic and autoimmune diseases by mediating IL-10 production in regulatory B cells. *J Allergy Clin Immunol.* 2013;131(6):1674–82. 10.1016/j.jaci.2013.01.044 23534976

[46] Chauhan V Kuo B McNamee JP: Transcriptional benchmark dose modeling: Exploring how advances in chemical risk assessment may be applied to the radiation field. *Environ Mol Mutagen.* 2016;57(8):589–604. 10.1002/em.22043 27601323

[47] Papathanasiou MA Kerr NC Robbins JH: Induction by ionizing radiation of the gadd45 gene in cultured human cells: lack of mediation by protein kinase C. *Mol Cell Biol.* 1991;11(2):1009–16. 10.1128/MCB.11.2.1009 1990262 PMC359769

[48] Langen B Rudqvist N Spetz J: Non-targeted transcriptomic effects upon thyroid irradiation: similarity between in-field and out-of-field responses varies with tissue type. *Sci Rep.* 2016;6:30738. 10.1038/srep30738 27779251 PMC5078841

[49] Ghandhi SA Turner HC Shuryak I: Whole thorax irradiation of non-human primates induces persistent nuclear damage and gene expression changes in peripheral blood cells. *PLoS One.* 2018;13(1):e0191402. 10.1371/journal.pone.0191402 29351567 PMC5774773

[50] Lucas J Dressman HK Suchindran S: A translatable predictor of human radiation exposure. *PLoS One.* 2014;9(9):e107897. 10.1371/journal.pone.0107897 25255453 PMC4177872

[51] Myeloid Cytokines for Acute Exposure to Myelosuppressive Doses of Radiation (Hematopoietic Subsyndrome of ARS), Cytokine - Radiation Emergency Medical Management.[cited 2018 Jan 12]. Reference Source

[52] Zhao JZL Mucaki EJ Rogan PK: Matlab Code for “Predicting Exposure to Ionizing Radiation by Biochemically-Inspired Genomic Machine Learning”. *Zenodo.* 2018; [cited 2018 Feb 9]. Data Source 10.12688/f1000research.14048.1 PMC5981198 29904591

