# Supplementary material for: Predicting ionizing radiation exposure using biochemically-inspired genomic machine learning
Source: F1000Res. 2018 Jun 15;7:233. Originally published 2018 Feb 27. [Version 2] doi: 10.12688/f1000research.14048.2 (PMC5981198; doi:10.12688/f1000research.14048.2)
Supplement: Peer review history

# Peer review: Referee response for version 1

**DOI:** 10.5256/f1000research.15278.r33717

**Published:** 2018-05-30

**Authors:** Roel Quintens, Mohamed Mysara

**Affiliations:** 1 Radiobiology Unit, expert group Interdisciplinary Biosciences, Institute for Environment, Health and Safety, Belgian Nuclear Research Centre, Mol, Belgium; 2 Expert Group Interdisciplinary Biosciences, Institute for Environment, Health and Safety, Belgian Nuclear Research Centre, Mol, Belgium

**Summary**

 In this study, the authors have identified gene signatures for radiation dose prediction using machine learning methodologies based on publically available microarray results from human and murine samples (mostly lymphocytes) exposed to ionizing radiation. Their signatures have been independently validated showing a high specificity for dose estimation. The authors have used a novel method, based on the concept of minimum redundancy maximum relevance. This generated signatures which often contained genes that had not previously been identified as potential radiation biomarkers. In all, this is a well-conducted study with relevance to the field. However, we do have some comments/questions/remarks, as outlined below.

**Introduction**

- Several other studies have applied machine learning methodologies to identify predictive radiation exposure biomarkers. Some of these have been reviewed in Hall *et al*., Mut Res 2017, Supplementary Table 3.5.1.1.
- Another important aspect of gene expression is alternative splicing, which also occurs in response to ionizing radiation (e.g. Sprung *et al.*, PLoS ONE 2011; Forrester *et al.*, PLoS ONE 2012; Macaeva *et al.,* Sci Rep 2016). The latter study also showed for the first time the suitability of exon signatures as sensitive radiation biomarkers, and highlights the importance of prior knowledge at the exon level for subsequent primer- or probe-based assays (e.g. qRT-PCR). This may be discussed.

 

**Methods**

- In the data Pre-processing “Rows and columns of microarray data that are less than 95% complete were removed and any remaining missing values were imputed using the nearest-neighbor algorithm” How many rows and columns were removed, and on which basis was the 95% threshold selected. Also, what is the effect of the nearest-neighbor algorithm on the data "over-fitting". Is it possible to perform PCA on the data after removing any row/columns with less than 100% completeness and compare to the currently presented approach (95% removal and filling the remainder of the missing data)? This would allow the visualization of the effect of the proposed methodology on the segregation between the various records.
- Only genes common to all datasets were retained. Does that mean common between mouse and human datasets? How were aliases identified?
- The second step in the process is the selection of genes based on a non-exhaustive list of publications. Why was this necessary if the mRMR method for feature selection was applied?
- I particularly like the idea of performing quantile normalization after feature selection. Is this something that has been published before? Can the authors speculate (or maybe even compare) about the performance of their method on pre-normalized datasets?
- Concerning the method used for the “Validation of models”, I would think this approach would be more vulnerable towards the test/training dataset. What would occur to the accuracy when doing the normalization over-all of the data? Would the accuracy change drastically? Is it possible to extend the testing to cover additional data?
- Many datasets exist on human PBMCs/whole blood irradiated with a range of doses. Why were these datasets not considered for this study while lymphoblastoid cell lines were?
- It would be helpful to have a comparison of model performance with that of “traditional” machine learning methods, as used in some of the indicated references.

 

**Results **

- “We discovered radiation gene signatures using the microarray data of human and mouse peripheral blood samples and human lymphoblastoid cell lines, which were validated either according to signature.” Were the human lymphoblastoid cell line and prepheral blood samples grouped together in one model? If so, would it be possible to visualize how the expression data of the shortlisted genes for each data type separately (using PCA for example)?
- Can the authors comment on their observation that signatures derived from both murine datasets are not very similar? Apart from “noise, or intrinsic differences in the datasets”, could it possibly also be a consequence of the method used, i.e. mRMR in which low mutual information genes are selected? Based on Fig. 2 and 6 it seems that genes with higher mutual information in general have higher frequencies. Which seems logical.
- The authors state that Ms4a1 appears less frequently than Glipr2. However, from the sizes of the circles, Ms4a1 seems to appear more frequently than Glipr2. Please verify this statement.
- Are genes in Tables 1 and 2 ranked according to their frequency, importance,…?
- How do the authors explain the low frequencies of human signature genes (Fig. 6), compared with murine (Fig. 2)?
- Likewise, can the authors explain the large number of genes with low mutual information in the human signature (23 out of 26 <0.4), compared with the murine signature (4 out of 33 <0.4).
- Although I like the idea of mRMR, it is somewhat counterintuitive to have genes with little mutual information to be important for dose prediction. This seems to be confirmed by the fact that the compositions of human signatures are dominated by genes with high mutual information (in fact, these are all well known p53-dependent genes which appear in a high number of published radiation signatures).

 

**Discussion**

- I understand the advantage of small signatures in terms of practicality. However, in case of a real emergency, in which individuals have been irradiated without good knowledge about the exact time since exposure, larger gene signatures may provide the additional benefit of having different dynamics per gene. This may help to also predict not only the dose, but also the time since exposure. Furthermore, one-gene signatures may suffer from higher variability among the population compared to larger gene signatures.
- I believe results from other, similar studies may be briefly situated in the introduction/discussion.

We have read this submission. We believe that we have an appropriate level of expertise to confirm that it is of an acceptable scientific standard.

## References

[1] HallJJeggoPAWestCGomolkaMQuintensRBadieCLaurentOAertsAAnastasovNAzimzadehOAzizovaTBaatoutSBaseletBBenotmaneMABlanchardonEGuéguenYHaghdoostSHarms-RinghdahlMHessJKreuzerMLaurierDMacaevaEManningGPernotERavanatJLSabatierLTackKTapioSZitzelsbergerHCardisE: Ionizing radiation biomarkers in epidemiological studies - An update. *Mutat Res*.771: 10.1016/j.mrrev.2017.01.001 59-84 10.1016/j.mrrev.2017.01.001 28342453

[2] SprungCNLiJHovanDMcKayMJForresterHB: Alternative transcript initiation and splicing as a response to DNA damage. *PLoS One*.2011;6(10) : 10.1371/journal.pone.0025758 e25758 10.1371/journal.pone.0025758 22039421PMC3198437

[3] ForresterHBLiJHovanDIvashkevichANSprungCN: DNA repair genes: alternative transcription and gene expression at the exon level in response to the DNA damaging agent, ionizing radiation. *PLoS One*.2012;7(12) : 10.1371/journal.pone.0053358 e53358 10.1371/journal.pone.0053358 23285288PMC3532210

[4] MacaevaESaeysYTaburyKJanssenAMichauxABenotmaneMADe VosWHBaatoutSQuintensR: Radiation-induced alternative transcription and splicing events and their applicability to practical biodosimetry. *Sci Rep*.2016;6: 10.1038/srep19251 19251 10.1038/srep19251 26763932PMC4725928

# Response

**Published:** 2018-06-09

**Authors:** Peter Rogan

**Affiliations:** University of Western Ontario, Canada

- Regarding other studies that identify predictive radiation exposure biomarkers, we have now added text citing these and other studies in  paragraph 1 of the introduction of version 2 of our paper.
- We investigated whether alternative splicing in response to ionizing radiation might affect the expression values that were used in training or validation of machine learning models we derived. These values could theoretically be distorted if the hybridization probes used to quantify gene expression in these microarray data were predominantly located in cassette exons. We carefully analyzed the probes on one of the most prominent genes in our signatures: *DDB2*, which consists of 10 exons in total. Out of the three human datasets used for deriving signatures, GSE6874[GPL4782] lacked information about the location of its *DDB2* probe. However, the *DDB2* probes in GSE10640[GPL6522] and GSE1725 were located in exons 8-10. According to UCSC Genome browser, the only transcript variants (NM_001300734.1, mRNA AB107039, and mRNA BC050455) involved skipping or fusing exons 3-7. Thus, in this case, alternative splicing does not affect our results, since the *DDB2* probes avoid alternatively spliced exons. In general, probes seem to be designed to avoid alternatively spliced regions. Although we have not verified this for all the genes in our signatures, we speculate that taking an average over multiple probes reduces any potential affect of alternative splicing.
- The nearest neighbor analysis was performed to avoid inclusion of genes or individuals with sparse data in the analysis. We conservatively selected  a threshold of 95% completeness to ensure that the original source data were reliable. At this threshold, none of the  rows or columns or each of the datasets we used for deriving signatures (GSE1725, GSE6874[GPL4782], GSE10640[GPL6522], GSE10640[GPL4783], GSE10640[GPL6524]) were removed (based on genes common among all datasets). The effect of nearest neighbours on overfitting was  minimal. Upon restriction to the set of genes available for feature selection, there were no missing values in GSE1725 and GSE6874[GPL4782], a single missing value in GSE10640[GPL6524] and GSE10640[GPL6522], and 62 missing values in GSE10640[GPL4783]. However, in GSE10640[GPL4783], of the genes available for feature selection, only four genes contained at least one missing expression value: *Rad51*, *Ptpre*, *Gadd45a*, and *Pola1*. None of these genes are among the 33 most frequently appearing genes of our murine signatures (see Figure 2). (Recall that GSE10640[GPL4783] is a murine dataset). Additionally, overfitting is mitigated by validation using independent datasets. The nearest neighbor source data occurs before model development, so the model is not fit at that point.
- Regarding the questions about genes common to all datasets being retained: 1) Mouse and human signatures were derived separately. So only genes common to all human datasets were available for selection in deriving human signatures and only genes common to all mice datasets were available for selection in deriving mouse signatures. 2) Our scripts did not take gene name aliases into account. It is therefore possible that genes that were left out of the analysis because they were indicated by different names in different datasets.
- The initial list of publications we consulted may not have been complete, but it was the result of an extensive search.  We were concerned that mRMR method applied to all genes without independent experimental support would result in Type II errors. We wanted to identify the key genes from the large volume of peer-reviewed work implicating various genes in radiation response. This hypothesis based study was not designed to discover novel genes, whose putative role in radiation response was unproven or unknown. We have automated the initial feature selection procedures using expression of all (including non-coding) genes, but this is beyond the scope of our efforts.  The discovery strategy would require statistical correction for the likelihood of incorrectly rejecting a null hypothesis due to multiple comparison testing.
- Regarding quantile normalization after feature selection, this is the first time that we have used this approach.  We attempted this because our initial efforts to derive signatures from pre-normalized data were unsuccessful (poor performance). Since we do not use the expression values for the majority of genes, there was no compelling reason to   normalize across the entire set of expressed genes. We are not aware of other previous efforts, but cannot exclude this possible. We did not perform an exhaustive literature search for the method that we employed, since the universe of potential applications is nearly limitless.
- We speculate that if quantile normalization is done *before *feature selection, then the reduction in dynamic range in the transformed data may  lead to the derivation of poorer signatures.To give a more quantitative answer, we took the top performing signature in the Y5 Supplementary file with respect to log loss ( *DDB2 GTF3A* *TNFRSF10B*) and re-validated with normalization over all data instead of just over genes in the signature; misclassification error was 2% higher and the goodness of fit was 4 cGy higher (log loss remained approximately the same). Interestingly, normalization performed over all of the data does not appear to have significant effects on the performance of our signatures. To “extend the testing to cover additional data,” it would be necessary to renormalize the initial set of genes to include these “additional genes”. If no other genes are to be included, only additional samples, then the expression values of the additional samples would need to be renormalized.
- Regarding other human datasets of irradiated PBMCs, in the feature selection stage, we specifically required datasets with large numbers of samples. The three human datasets we used for feature selection (GSE6874, GSE10640, GSE1725) were the largest ones we found deposited in GEO. In particular, GSE1725, the dataset with cell lines derived from patients, was the largest dataset available to us, containing 110 samples (171 samples in total, but 61 are UV irradiated). At the earliest stage of our project, we specifically chose datasets that maximized the number of common genes represented among them. This requirement enabled us to validate our signatures by either traditional or k-fold approaches.   Genes available for feature selection must be present in all datasets in order for validation to work.  We were surprised to find many datasets where key genes in our models were missing from expression data (see the last section on partial body irradiation data analysis in the results section of the revised version 2 of this paper).
- Regarding comparison of model performance with other traditional machine learning methods, we have used these methods (eg. random forest, SVMs, decision trees) in previous gene expression studies (see reference 13 of version 1 which is reference 18 of version 2). The improved performance we describe here cannot be attributed to the specific model building approaches.
- Regarding grouping of lymphoblastoid and PBL samples, models were always trained on one dataset at a time; they were not combined.
- The dissimilarity of the murine datasets may be related to their use of different microarray platforms and were collected at different times. It is difficult to tease out the intrinsic differences of the datasets from the performance of the methods.
- Regarding Ms4a1 and Glipr2, we verify the reviewers' observation. We corrected the density plot during preparation of the paper, but inadvertently neglected to make the corresponding change to the text.  We have replaced *Glipr2* with *Ccng1* or *Eif2ak4*.
- Regarding ranking of genes in Tables 1 and 2,  signatures derived using BSFS and CSFS list the genes according to mRMR rank. For signatures derived using FSFS, the genes would be listed according to the order in which they were selected. So in this sense, they are indeed ranked according to importance.
- Regarding the low frequencies of human vs murine signature genes, in the descriptions of these figures, we mention that “Frequencies are first scaled within and then between datasets to ensure values between 0 and 1.” Human signature gene frequencies appear suppressed because *DDB2* and *GADD45A* in particular were represented more frequently than any other gene by a large margin.
- Regarding the numbers of genes with low mutual information in the human vs murine signatures, it may be relevant that expression data are not adjusted for either white blood cell count or body mass. We speculate that the gene expression response in the human samples reflects lower numbers of radiation exposed cells. This could dampen the signals and mutual information with radiation dose compared to the murine response.
- Regarding the idea that it would be counterintuitive to have genes with low mutual information as important for dose prediction, it is true that signatures are dominated by genes whose expression values share high mutual information with dose. The purpose of mRMR is to make sure that we do not overlook the genes whose expression values may encode information that is not present in the genes whose expression values have high mutual information with dose, which is why sometimes genes with lower mutual information may appear as frequently or even a bit more frequently than genes with higher mutual information.
- Regarding the single gene signature results, we agree that single gene signatures are more susceptible to extrinsic sources of variation unrelated to radiation exposure. However, simpler signatures may be necessary under laboratory conditions that limit the amount or complexity of testing, e.g. space radiation assays performed by astronauts.

# Peer review: Referee response for version 1

**DOI:** 10.5256/f1000research.15278.r31994

**Published:** 2018-05-30

**Authors:** Michael D. Story, Liang-hao Ding

**Affiliations:** 1 Department of Radiation Oncology, University of Texas Southwestern Medical Center, Dallas, TX, USA

The authors are absolutely correct that in the event of a radiological accident blood is likely to be the most likely source for analytical materials that would reflect a radiation exposure. Given the analytical approach taken we tested this using two sets of data from normal human lung epithelial cells (HBECs) irradiated at multiple doses by γ-rays but also with Fe particles such as those found in the deep space environment. (Reference 8 in this manuscript)

 We were interested in two things: 1) Would the results from lymphoid cells translate to epithelial cells? There is sufficient evidence in the literature to suggest signatures created with lymphoid cells are poor at predicting radio response in cells from tissue. 2) Would the results from γ-ray exposures translate to something more exotic like Fe particles which have discriminating gene sets that are both common to γ-ray and Fe particles as well as unique to the radiation type.

 We chose the 10 gene signature derived from GSE6874 and validated against GSE10640. Given the limited size of our sample set, we were surprised to see Prediction Accuracies of 73% for γ-rays and 83% for Fe particle irradiation when examining the normal HBEC cells; and 85 and 76%, respectively, for a genetically manipulated HBEC (p53 knockdown, KRAS mutant over-expressing) cell line.

 We look forward to testing this approach in genetically diverse tumor cells.

We have read this submission. We believe that we have an appropriate level of expertise to confirm that it is of an acceptable scientific standard.

# Response

**Published:** 2018-06-09

**Authors:** Peter Rogan

**Affiliations:** University of Western Ontario, Canada

Thank you for your efforts  to review our article and evaluate our software. We were excited about the significant results you obtained using the human signature on expression data from irradiated lung epithelial cells, and using the models to detect evidence of Fe particle radiation.

# Peer review: Referee response for version 1

**DOI:** 10.5256/f1000research.15278.r33291

**Published:** 2018-05-14

**Authors:** Daniel Oh

**Affiliations:** 1 University of North Carolina at Chapel Hill, Chapel Hill, NC, USA

Well written article. Good analysis of available gene expression data to create gene signatures for ionizing radiation exposure. Good discussion of the identified genes' functions and roles in radiation response. Conclusions are well supported by the results. Hopefully the analysis and genes identified in this study will be incorporated and/or validated in future studies examining prediction of radiation exposure.

I have read this submission. I believe that I have an appropriate level of expertise to confirm that it is of an acceptable scientific standard.

# Response

**Published:** 2018-06-09

**Authors:** Peter Rogan

**Affiliations:** University of Western Ontario, Canada

Thank you for your kind comments. We agree that the approach and software  should be useful for future studies of human radiation exposures. We are particularly motivated to apply multiclass SVMs, which were highly accurate in the murine dataset,  to the analysis of a large set of radiation oncology patients exposed to different radiation doses.
